# Evaluating the effectiveness and cost-effectiveness of Dementia Care Mapping™ to enable person-centred care for people with dementia and their carers (DCM-EPIC) in care homes: study protocol for a randomised controlled trial

**DOI:** 10.1186/s13063-016-1416-z

**Published:** 2016-06-24

**Authors:** Claire A. Surr, Rebecca E. A. Walwyn, Amanda Lilley-Kelly, Robert Cicero, David Meads, Clive Ballard, Kayleigh Burton, Lynn Chenoweth, Anne Corbett, Byron Creese, Murna Downs, Amanda J. Farrin, Jane Fossey, Lucy Garrod, Elizabeth H. Graham, Alys Griffiths, Ivana Holloway, Sharon Jones, Baber Malik, Najma Siddiqi, Louise Robinson, Graham Stokes, Daphne Wallace

**Affiliations:** Faculty of Health and Social Sciences, Leeds Beckett University, Leeds, LS1 3HE UK; Leeds Institute for Clinical Trials Research, University of Leeds, Leeds, LS2 9PH UK; Leeds Institute of Health Sciences, University of Leeds, Leeds, LS2 9LJ UK; Wolfson Centre for Age Related Diseases, Kings College London, London, UK; Psychological Services, Oxford Health NHS Foundation Trust, Oxford, OX3 7JX UK; School of Dementia Studies, University of Bradford, Bradford, BD7 1DP UK; University of Technology, Sydney, NSW 2007 Australia; Bradford District Care Foundation Trust, Bradford, UK; Institute for Aging and Health, University of Newcastle, Newcastle upon Tyne, NE1 7RU UK; Bupa, London, UK

**Keywords:** Agitation, Care homes, Cluster-randomised controlled trial, Dementia, Dementia Care Mapping, Person-centred care

## Abstract

**Background:**

Up to 90 % of people living with dementia in care homes experience one or more behaviours that staff may describe as challenging to support (BSC). Of these agitation is the most common and difficult to manage. The presence of agitation is associated with fewer visits from relatives, poorer quality of life and social isolation. It is recommended that agitation is treated through psychosocial interventions. Dementia Care Mapping™ (DCM™) is an established, widely used observational tool and practice development cycle, for ensuring a systematic approach to providing person-centred care. There is a body of practice-based literature and experience to suggests that DCM™ is potentially effective but limited robust evidence for its effectiveness, and no examination of its cost-effectiveness, as a UK health care intervention. Therefore, a definitive randomised controlled trial (RCT) of DCM™ in the UK is urgently needed.

**Methods/design:**

A pragmatic, multi-centre, cluster-randomised controlled trial of Dementia Care Mapping (DCM™) plus Usual Care (UC) versus UC alone, where UC is the normal care delivered within the care home following a minimum level of dementia awareness training. The trial will take place in residential, nursing and dementia-specialist care homes across West Yorkshire, Oxfordshire and London, with residents with dementia. A random sample of 50 care homes will be selected within which a minimum of 750 residents will be registered. Care homes will be randomised in an allocation ratio of 3:2 to receive either intervention or control. Outcome measures will be obtained at 6 and 16 months following randomisation. The primary outcome is agitation as measured by the Cohen-Mansfield Agitation Inventory, at 16 months post randomisation. Key secondary outcomes are other BSC and quality of life. There will be an integral cost-effectiveness analysis and a process evaluation.

**Discussion:**

The protocol was refined following a pilot of trial procedures. Changes include replacement of a questionnaire, whose wording caused some residents distress, to an adapted version specifically designed for use in care homes, a change to the randomisation stratification factors, adaption in how the staff measures are collected to encourage greater compliance, and additional reminders to intervention homes of when mapping cycles are due, via text message.

**Trial registration:**

Current Controlled Trials ISRCTN82288852. Registered on 16 January 2014.

Full protocol version and date: v7.1: 18 December 2015.

**Additional file:**

The online version of this article (doi:10.1186/s13063-016-1416-z) contains supplementary material, which is available to authorized users.

## Background

A third of people who have dementia reside in a care home [1] and at least two thirds of people living in care homes have dementia [2]. Of the people living with dementia, up to 90 % experience one or more behaviours that staff may describe as ‘challenging’ to support (BSC), during the course of their condition. BSC include behaviours such as agitation, aggression, restlessness, hallucinations, delusions, depression, anxiety and apathy [3]. The most common of these, with reported prevalence rates of over 60 % in nursing home residents with dementia, is agitation [4, 5], which includes a cluster of extremely problematic behaviours such as aggressive and physically non-aggressive behaviours and verbal agitation [6]. The presence of agitation in a person with dementia is associated with fewer visits from relatives, poorer quality of life [7] and social isolation [8]. Furthermore, it puts the person who is agitated at risk of triggering responses from other residents [9], causing potential serious risk of harm not only to the person who is agitated, but to other residents and staff. Agitation and other BSC are not an inevitable consequence of dementia, they reflect an expression of unmet needs by a person with dementia in response to poor quality care [10–12]. It is recognised that the presence of agitation in individuals with dementia in care home settings is associated with poorer levels of organisational aspects of care and the care culture [11]. It is, therefore, recommended that agitation is treated through the use of psychosocial interventions that address the quality of care practice [10, 13].

Person-centred care is an effective psychosocial approach in dementia care [14] and is considered a best practice method for reducing agitation and other BSC [13]. Person-centred care means providing a supportive social environment within a care setting where people with dementia are valued, treated as individuals, and staff are encouraged to see the world from the person’s perspective [13, 15]. Raising staff knowledge, skills and confidence levels around person-centred ways of working with BSC is, therefore, a national priority area [16–18]. Training staff in person-centred approaches has been found to be effective in improving the delivery of person-centred care [19, 20]. However, whilst effective person-centred care training can produce immediate practice benefits, evidence suggests that alone it might not sustain change over time [19–22] and additional support is required in order to facilitate sustained benefits [23] over an extended period of time [24].

Dementia Care Mapping™ (DCM™) [25, 26] is an established and widely used intervention, directed at care homes, for ensuring a systematic approach to providing individualised person-centred care, and is recommended by the National Institute for Clinical Excellence/Social Care Institute for Excellence (NICE/SCIE) [13]. DCM™ is an observational tool, set within a practice development cycle, which includes five phases: (1) briefing, (2) observation, (3) analysis, (4) feedback, and (5) action planning. This cycle is repeated every 4–6 months to monitor and revise action plans. Once initial training and skills development in the method are completed, those trained to use DCM™ (mappers) are able to conduct these practice development cycles (mapping) independently. This means that DCM™ requires no external input over the long term and is, therefore, potentially less resource intensive and more likely to be readily implemented in real-world dementia practice than other interventions [27]. Whilst DCM™ has been used in dementia care for nearly 20 years, including implementation in care home settings [28–32], and has strong face validity within the practice field [33], there is limited robust evidence of its efficacy in relation to clinical outcomes such as reduction of BSC. Practice implementation suggests that the benefits of DCM™ include the improvement of well-being in service users [34–36] and helping staff see care from the point of view of the person with dementia, leading to evidence-based feedback and action planning that motivates staff and helps them to feel more confident to implement person-centred care [32, 33].

To date there are only five published studies that examine the benefits of using DCM™ for improving clinical outcomes. A Dutch pilot study [37] utilising a One-Group Pretest-Posttest design found DCM™, used alone, reduced verbal agitation and anxiety in people with dementia and improved care staff feelings of connection with clients. An Australian pilot study [38] in three care homes, employing a Pretest-Posttest design, found improvements in the quality of staff interactions and reductions in agitation and depression through the use of DCM™. There are three full RCTs of the effectiveness of DCM™ published to date. A cluster RCT conducted in 15 care homes with 289 residents (loss to follow-up of 18 % at 10 months) in Australia [14] (UC = 5, UC + person-centred care training = 5, UC + DCM = 5), found that at 10 months post randomisation, DCM™, when used alone was associated with significantly reduced agitation and falls among residents with dementia compared to UC. A Norwegian cluster RCT [39] in 15 care homes (5 = control group, 5 = person-centred care framework implementation, 5 = DCM) and with 446 residents (loss to follow-up of 29 % at 10 months) found a significant reduction in neuropsychiatric symptoms as measured by the Neuropsychiatric Inventory (NPI) and on the NPI sub-scales of agitation and psychosis compared to controls. It also found a significant improvement in quality of life, compared to controls after 10-month follow-up. However, both the Norwegian and the Australian study had a follow-up period of only 10 months, limiting the potential for impact given the length of time that changes within practice can take to implement and thus potential benefits to be observed. Additionally, both trials had explanatory designs involving researcher-led cycles of DCM™ with variable degrees of input from trained care home staff. This restricts generalisability of the results to usual implementation of DCM™ in care practice, which is practitioner-led. A cluster RCT study in 34 units, from 11 care homes in the Netherlands [40], with 434 residents (loss to follow-up 35 % at 12 months) found no difference in residents’ agitation between the DCM™ intervention and control homes. However, staff in the intervention group reported significantly fewer negative emotional reactions and significantly more positive reactions towards people with dementia over time. This trial reported potential intervention fidelity issues in the DCM™ care homes, indicating less than desirable implementation of the intervention in some of the clusters. A limitation of all three RCT studies is that they were exploratory studies and each only included two full cycles of DCM™ before final follow-up, reducing the time for potential change and impact to be realised.

Despite their limitations, these studies provide promising data on the effectiveness of DCM™ in Australian, Norwegian and Dutch care home settings. They do not provide, however, a robust evaluation of effectiveness of DCM™ in UK settings. In particular, the Australian study used DCM™ alone rather than alongside person-centred care training, which is recommended in DCM implementation guidelines [41]. This reflects the Australian context at the time of the study, where staff access to person-centred care training was the exception rather than assumed good practice, but it highlights the lack of comparability of Usual Care between UK and non-UK care homes. In addition, there are distinct funding models of dementia care across countries and, therefore, the economic evaluation data from these RCTs are not directly applicable to a UK context. Therefore, a definitive RCT of DCM™ in the UK, building on previous work, is needed to inform the delivery of person-centred dementia care within UK care homes. The additional knowledge to be gained from this trial, beyond that within research conducted to date, is that:

- It will reflect conditions of DCM™ implementation in usual practice, being a pragmatic trial, compared to the explanatory designs of previous trials; in particular with care home staff rather than researcher led cycles of DCM™ implementation. The study design, size and statistical power will permit definitive conclusions to be drawn regarding the efficacy of DCM™ as an intervention in care home settings
- Three cycles of the DCM™ intervention will be implemented and follow-up will be over a period of 16 months; considerably longer than in previous trials where follow-up has been a maximum of 10 months. This is beneficial since some practice changes, for example to underlying care culture, are likely to require time to implement and, therefore, a longer follow-up period is necessary to investigate any such effects
- This trial will conduct a full economic evaluation utilising a pragmatic trial design and, therefore, will be able to offer a definitive position on cost-effectiveness. Only one of the previous trials conducted an economic evaluation and given its explanatory design, these findings cannot be confidently generalised
- The trial design builds on that of the three previous explanatory trials, meaning its design is optimal for assessing efficacy of DCM™ as an intervention in care home settings

## Aims and objectives

The aim of the trial is to evaluate the clinical and cost-effectiveness of DCM™ in addition to Usual Care (UC) compared to UC alone for people with dementia living in care homes in the UK.

### Primary objective

To determine if DCM™ plus UC (i.e. the intervention) is (1) more effective in reducing agitation as measured by the total Cohen-Mansfield Agitation Inventory (CMAI) score and (2) more cost-effective than UC alone (i.e. the control), 16 months following randomisation of care homes.

### Secondary objectives

Secondary objectives are to investigate the effectiveness of the intervention at 6 and 16 months post randomisation in: (1) reducing BSC in residents over time as measured by the CMAI and the NPI, (2) reducing the use of antipsychotic and other psychotropic drugs, (3) improving resident mood and quality of life, (4) improving staff well-being and role efficacy, and (5) improving the quality of staff/resident interactions over time, as measured by the Quality of Interactions Schedule (QUIS). It will also explore (6) the safety profile of the intervention as assessed by the number and types of adverse events, (7) any differential predictors of the effects of an intervention, and (8) the process, challenges, benefits and impact of implementing the intervention.

## Methods/design

### Design

**Fig. 1:**
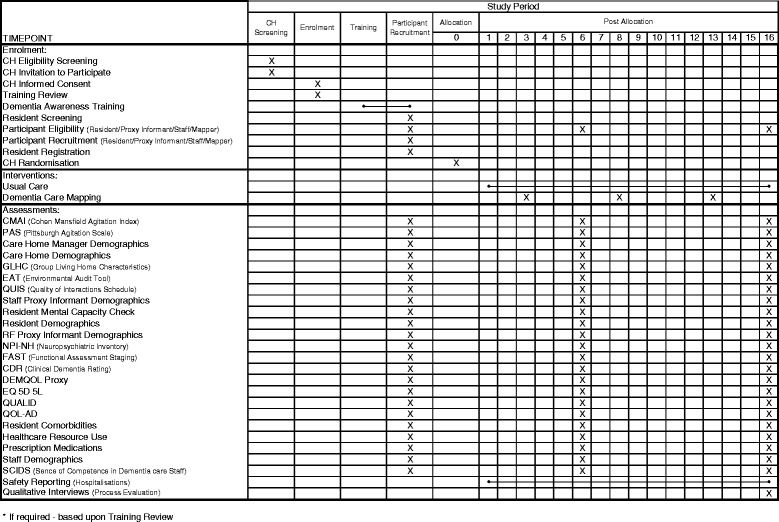
Standard Protocol Items: Recommendations for Interventional Trials (SPIRIT) checklist

This trial has been designed to be a pragmatic, multi-centre, cluster-randomised controlled trial of DCM™ plus UC versus UC alone. There will be four types of trial participants: care homes, residents, their relative/friend and care home staff members. The trial will take place in residential, nursing and dementia UK care homes across West Yorkshire, Oxfordshire and London. From these areas, 50 care homes will be recruited from a random sample, within which 750 residents, their relative/friend (where eligible) and all eligible, consented, care home staff will be registered. Following participant identification and consent, baseline assessments will be undertaken and then the care homes will be randomised in a ratio of 3:2 to receive intervention or control. Outcome measures will be obtained at 6 and 16 months following randomisation. Additional to the primary analysis, there will be an integral cost-effectiveness analysis and a process evaluation. Figure 1 outlines the schedule for all trial activities.

### Inclusion and exclusion criteria

#### Care home criteria

A care home meeting all of the following criteria at screening will be eligible for this trial:

- Has a sufficient number of permanent dementia (based on a formal diagnosis or Functional Assessment Staging of Alzheimer’s Disease (FAST) [42] score of 4+) residents eligible to participate in the study in order to achieve a minimum of 10 residents registered to take part
- Has a manager or nominated person agreeing to sign up to the trial protocol as research lead for the duration of the project?
- Agrees to release staff for DCM™ training and subsequent mapping processes
- Is within the catchment area

A care home meeting any of the following criteria will not be eligible for this trial:

- In the view of the research team, is not suitable for inclusion due to being subject to Care Quality Commission enforcement notices, admission bans or relevant moderate or major Care Quality Commission compliance breaches
- Is receiving other special support for specific quality concerns, such as being currently subject to, or have pending, any serious safeguarding investigations, or receiving voluntary or compulsory admissions bans, is in receipt of local commissioning special support due to quality concerns
- Has used DCM™ as a practice development tool within the 18 months prior to randomisation or is planning to use DCM™ over the course of trial involvement
- Is taking part, has recently taken part in, or is planning to take part, in another trial that conflicts with DCM™ or with the data collection during the course of their involvement in the trial

#### Resident criteria

Residents meeting all of the following criteria at screening will be eligible for this trial:

- Is a permanent resident within the care home – defined as a person residing in the care home and not present for receipt of respite or day-care only
- Has a formal diagnosis of dementia or score 4+ on FAST [42] as rated by the home manager or another experienced member of staff
- Is appropriately consented (in accordance with the Mental Capacity Act [43] and clinical trial guidance on informed consent [44–46])
- Has an allocated member of staff willing to provide proxy data
- Has sufficient proficiency in English to contribute to the data collection required for the research

Residents meeting any of the following criteria will not be eligible for this trial:

- Is known by the care home manager and/or relevant senior staff member to be terminally ill, e.g. formally admitted to an end of life care pathway
- Is permanently bed-bound/cared for in bed

#### Staff criteria

1. *Proxy informant*: to be eligible to provide proxy data staff must meet all of the following criteria:
  Be a permanent or contracted member of staff
  Know the resident well, as assessed by their key worker status and/or the judgement of the home manager
  Staff are ineligible if they meet any of the following criteria:
  Working in the home as agency or bank staff
  Have consented to be one of the home’s trained DCM™ mappers
  Have acted as a nominated consultee for any residents in the trial
2. Provision of staff measures: staff meeting all of the following criteria will be eligible to provide data on the staff measures:
  Is a permanent, contracted, agency or bank member of staff at time of data collection
  Provides consent to providing data for the trial through return of the Staff Measures booklet
  Has sufficient proficiency in English to contribute to the data collection required for the research
  Staff meeting the criterion below will not be eligible to provide data on the staff measures for this trial:
  Be acting as a nominated consultee for any residents participating in the trial
3. *Dementia Care Mapper*: to be eligible to undertake this role staff must:
  Be a permanent or contracted member of staff
  Have the right skills and qualities to be a mapper as assessed by the home manager in accordance with guidance provided by the research team, and
  Provide consent to becoming a mapper, to implementing the DCM™ process as per the research protocol and to participate fully in the process evaluation
  Staff who meet any of the following criteria are not eligible to be a mapper:
  Work in the home as agency or bank staff
  Have acted as a nominated consultee for any residents participating in the trial
  Provide proxy data for any residents participating in the trial

#### Relative/friend criteria

To be eligible to provide proxy data about a resident, relatives/friends must:

- Have visited the resident on a regular basis over the past month (i.e. at least once per week)
- Be willing to provide data at a time convenient to them
- Have sufficient proficiency in English to contribute to the data collection required for the research

Eligibility waivers to inclusion and exclusion criteria are not permitted.

### Recruitment

#### Care home recruitment

Recruitment will begin with an initial eligibility screening (24 beds or larger in order to ensure that minimum cluster size is likely to be achieved and providing care to older people) of all care homes in the recruitment hub areas (West Yorkshire, Oxfordshire, London) via publicly available information, after which care homes will be approached from those deemed eligible using two-stage sampling. In the first stage, catchment areas within each recruitment hub, defined by postcode prefix for West Yorkshire, boroughs for London and geographical area for Oxfordshire, will be selected in rotation. In the second stage, all the care homes within catchment areas will be randomly ordered and, at each rotation, a batch of 12 care homes from the catchment area will be sent invitation information by post. This method ensures geographical closeness of care homes approached for recruitment at the same time, which will support multiple short recruitment visits to be undertaken with minimised travel. Sampling and care home approach will be staggered across the recruitment period.

Researchers will contact homes invited to participate by telephone after sending an initial postal invitation. For interested care homes, the researcher will complete initial eligibility screening via telephone and then visit the care home to determine full eligibility and complete the recruitment process. Once all the care homes within a catchment area batch have been contacted and a decision regarding participation made, the researcher will move onto the next batch from the next catchment area until sufficient homes have been recruited. In this way, it is intended that the care home sample will be representative of the entire region sampled, and that any deviations from this will be known and can be adjusted for. The target is for four homes to be recruited per month across the whole trial.

Given that DCM™ is designed to be used alongside training in person-centred dementia care, all care homes will be audited using a training audit tool designed by the research team. This will ensure that each home in the trial meets at least minimum dementia training levels defined in terms of both content of the training and proportion of staff trained (minimum 20 % of direct care staff). If the training audit finds that a home has not provided staff with a minimum level of dementia awareness training, staff will be provided with a half-day dementia awareness course. Based on existing published data [45], we expect up to 20 % of homes to require this dementia awareness package.

#### Resident recruitment

Following the training audit, the researcher will meet with the care home manager and/or relevant senior staff member to identify all eligible residents to be approached to take part in the trial. All residents will be reviewed for eligibility by the researcher through discussions with the manager, whilst maintaining anonymity. All eligible residents will then be approached to participate. It is expected that approximately 15 residents will be recruited at each participating care home. Reasons for ineligibility will be recorded.

#### Staff recruitment

Care home staff can be recruited into four roles: (1) as proxy informants, providing data about resident participants, (2) as a staff participant providing data about him or herself, (3) as a mapper who will be responsible for implementing the intervention, and (4) as a nominated consultee (see informed consent). Role 4 is mutually exclusive from all other roles, so any staff member recruited as a nominated consultee will not be permitted to undertake any other role within the trial. Roles 1 and 3 are also mutually exclusive, so staff recruited as mappers will not be permitted to act as proxy informants. Recruitment to all staff roles will occur at baseline and at subsequent time points where necessary due to withdrawal. For role 2, recruitment will take place at baseline and at each further data collection point, due to the expected annual turnover rates of staff in each care home.

#### Relative/friend recruitment

The resident (where possible), or the care home manager, will be asked to identify a relative or close friend of the resident, who visits at least once per week, to be approached to provide proxy data for the trial. The relative/friend will be contacted by post with information about the trial and asked to return a signed consent form to the care home if they agree to take part. The person providing proxy data may differ from the residents’ personal consultee, where one is appointed (see informed consent).

### Intervention

#### Dementia Care Mapping (DCM™)

DCM™ will be implemented according the standard procedures identified in the DCM™ 8 User’s Manual [26]. The intervention comprises training two care home staff in use of DCM™ followed by implementation of three full mapping cycles. Two eligible staff members will be identified as mappers in all homes prior to randomisation and will be consented at baseline. After completion of the standard 4-day DCM™ training course, the mappers will run briefing sessions 1–2 weeks prior to undertaking the mapping observations. During the briefing session, mappers will consult with staff about selection of appropriate residents to be mapped. Residents chosen for inclusion in mapping observations do not have to be trial participants and verbal consent from residents to be observed will be gained by the mappers ahead of mapping observations commencing. Mapping involves the mappers continuously observing between two and five people with dementia, over a period of four to six consecutive hours, in communal living areas only. The mappers will then analyse the data they collect and present it in a report that will be fed back to the staff team. During the feedback session, an action plan will be produced in collaboration with the staff team, which will detail areas that the home aims to improve, based on the DCM™ data. Progress on these actions is monitored during the next mapping cycle. The first cycle should be completed by approximately 3 months post randomisation and the second and third cycles at approximately 8 months and 13 months post randomisation, respectively. An expert mapper, who is a practitioner experienced in the implementation of DCM™, will provide support to each care home during completion of their first cycle of mapping in order to maximise intervention fidelity across all homes. Telephone/email support for DCM™ implementation will be available to all care homes thereafter through the DCM™ lead for the trial, if required. Mappers will be asked to complete and return data on mapping practice for all three mapping cycles including: information on numbers of staff in the care home receiving DCM™ briefing; number of hours of mapping; number of residents mapped; number of staff attending feedback sessions and number of action plans developed. Mappers will be provided with a standard reporting template to gather this information.

#### Usual Care

UC is defined as normal care delivered within the setting (as measured by training audit, a bespoke UC Questionnaire and the Care Home Context and Organisational Questionnaire). No restrictions will be imposed on current practices or on homes undertaking additional development or training as part of UC, with the exception of control arm homes being required not to implement DCM™ during their trial involvement period. Person-centred care is considered best practice within dementia care [13] and as such care homes are expected to provide staff with appropriate training to deliver care of this type [47].

### Registration and randomisation

Residents will be registered centrally with the Clinical Trials Research Unit (CTRU) at the University of Leeds after care home recruitment, the care home training audit, confirmation of eligibility, informed consent and collection of resident baseline data.

Once all residents within a care home have been registered, care homes will be randomised centrally at the CTRU to receive DCM™ + UC or UC, leading to two sources of clustering: cluster-randomisation and DCM™ treatment provision. The former occurs at randomisation (care homes are nested within treatment arms), the latter afterwards (care homes are partially nested within arms) so we anticipated that the clustering effect will vary across arms, and assumed a higher design effect in the intervention arm. Care homes will be randomised on a 3:2 basis. A computer-generated minimisation programme incorporating a random element will be used to ensure treatment arms are balanced for the following care home characteristics: (1) home/unit type (general residential/nursing, specialise in dementia care), (2) size (large at least 40, medium/small fewer than 40), and (3) provision of dementia awareness training by research team (yes, no), (4) recruitment hub (West Yorkshire, Oxfordshire, London). The latter was changed from prior use of DCM™ in the last 5 years as balancing the intervention across recruitment hubs was considered more important. Following randomisation, a member of the research team will inform the care home manager of their allocation and for those homes allocated to DCM™ + UC, the staff consented to take on the role of mappers will also be informed, so arrangements for attendance at DCM™ training can be made.

### Data assessments

Assessments will be undertaken at screening (prior to consent); baseline (prior to resident registration); 6 months post randomisation and 16 months post randomisation. Baseline data collection visits will be conducted over approximately 3 weeks in each home and follow-up visits over a 1–2-week period. Required data, assessment tools, collection time points and processes are summarised in Table 1.

**Table 1:** Summary of assessments

Assessment	Type	Method of completion	Timeline			
			Screening	Baseline	6 months	16 months
Care home eligibility	CRF	Researcher assessment	X			
Training review	CRF	Researcher assessment	X			
Dementia awareness training	CRF	Dementia awareness trainer	X			
Resident screening (demographics)	CRF	Researcher assessment	X			
Staff mapper screening	CRF	Researcher assessment	X			
Consent (staff mapper, resident (includes personal/nominated consultee), staff proxy informant, RF proxy informant)	Consent Form	Self-completion (witnessed)	X			X
Participant eligibility (staff mapper, resident, staff proxy informant, RF proxy informant)	CRF	Researcher assessment	X			X
Participant contact details (resident, staff proxy informant, RF proxy informant)	CRF	Researcher assessment	X			X
Cohen-Mansfield Agitation Index (CMAI) abridged	Questionnaire booklet	Independent researcher observations (R)		X	X	X
Pittsburgh Agitation Scale (PAS)	Questionnaire booklet	Independent researcher observations (R)		X	X	X
Care home manager demographics	Questionnaire booklet	Researcher interview (CM)		X	X	X
Care home demographics	Questionnaire booklet	Researcher interview (CM)		X	X	X
Group Living Home Characteristics (GLHC)	Questionnaire booklet	Researcher assessment (CH)		X	X	X
Environmental Audit Tool (EAT)	Questionnaire booklet	Researcher observations (CH)		X	X	X
Quality of Interactions Schedule (QUIS)	Questionnaire booklet	Researcher observations (R/S)		X	X	X
Staff proxy informant demographics	Questionnaire booklet	Researcher interview (SP)		X	X	X
Resident demographics	Questionnaire booklet	Researcher assessment		X	X	X
RF proxy informant demographics	Questionnaire booklet	Researcher interview (RF)		X	X	X
Cohen-Mansfield Agitation Index (CMAI)	Questionnaire booklet	Researcher interview (SP)		X	X	X
Neuropsychiatric Inventory (NPI-NH)	Questionnaire booklet	Researcher interview (SP)		X	X	X
Functional Assessment Staging (FAST)	Questionnaire booklet	Researcher interview (SP)		X	X	X
Clinical Dementia Rating (CDR)	Questionnaire booklet	Researcher interview (SP)		X	X	X
DEMQOL-Proxy	Questionnaire booklet	Researcher interview (SP/RF)		X	X	X^a^
EQ-5D-5 L	Questionnaire booklet	Researcher interview (SP/RF/R)		X	X	X^a^
QUALID	Questionnaire booklet	Researcher interview (SP/RF)		X	X	X^a^
QOL-AD	Questionnaire booklet	Researcher interview (R)		X	X	X
Resident comorbidities	Questionnaire booklet	Researcher assessment		X	X	X
Health care resource use	Questionnaire booklet	Researcher assessment		X	X	X
Prescription medications	CRF	Researcher assessment		X	X	X
Resident registration	Questionnaire booklet	Researcher assessment		X		X
Staff booklet	Questionnaire booklet	Self-completed (S)		X	X	X
General Health Questionnaire (GHQ-12)	Questionnaire booklet	Self-completed (S)		X	X	
Sense of Competence in Dementia care Staff (SCIDS) scale	Questionnaire booklet	Self-completed (S)		X	X	X
Safety reporting	CRF	Researcher assessment		Monthly following randomisation		
RUSAE Report	CRF	Researcher assessment		As highlighted.		
			Mapper training	Cycle 1	Cycle 2	Cycle 3
Mapper training	CRF	DCM™ trainer	X			
DCM™ adherence	Questionnaire booklet/CRF	DCM™ expert/ independent reviewer		X	X	X
DCM™ briefing summary	CRF	CH mapper		X	X	X
DCM™ feedback summary	CRF	CH mapper		X	X	X

Key: *CRF* Case Report Form, *CH* care home observations, *CM* care home manager, *DEMQOL-Proxy* Dementia Quality of Life measure – proxy version, *EQ-5D-5 L* EuroQol five dimensions, five levels, *QUALID* Quality of Life in Late-Stage Dementia, *QOL-AD* Quality of Life in Alzheimer’s disease measure, *R* resident, *RF* relative/friend proxy informant, *RUSAE* Related Unexpected Serious Adverse Event, *S* staff, *SP* staff proxy informant, X^a^ – only for relative/friend informants consented at baseline and still meeting eligibility criteria

### Outcomes

The primary outcome is agitation at 16 months following randomisation. The primary measure of agitation is the Cohen-Mansfield Agitation Inventory (CMAI) [48] rated by a staff member who knows the resident well. The Pittsburgh Agitation Scale (PAS) [49] and an adapted CMAI (see below), rated by an independent researcher not involved in any other data collection within that care home and blinded to allocation, will provide concurrent validity. This addresses the issue of potential bias of staff responses, based on the inability to blind them to allocation status.

There are a number of secondary outcomes each relating to residents, staff or care homes. Secondary outcomes at 6 and 16 months post randomisation relating to residents are: Neuropsychiatric Inventory (NPI) [50]; DEMQOL-Proxy, Quality of Life in Late Stage Dementia (QUALID) [51]; QOL-AD [52], EuroQol five dimensions, five levels (EQ-5D-5 L) [53]; the prescription and use of psychotropics, memantine, benzodiazepines and anti-depressants; and, safety reporting (serious adverse events (SAEs)). Secondary endpoints at 6 and 16 months post randomisation relating to staff are: General Health Questionnaire (GHQ-12) [54] and Sense of Competence in Dementia care Staff (SCIDS) scale [55]. Secondary endpoints relating to homes are: intervention fidelity (at 16 months) and Quality of Interactions Schedule (QUIS) [56] at 6 and 16 months.

### Outcome measures

#### Resident-related outcome measures

##### Agitation

**Cohen-Mansfield Agitation Inventory (CMAI)** [6, 48]:

The CMAI measures 29 agitated or aggressive behaviours [57] on frequency, using a seven-point scale (1–7) ranging from ‘never’ to ‘several times an hour’ based on behaviours over the previous 2 weeks. A total score is obtained by summing the 29 individual frequency scores (range 29–203). It has good psychometric properties [58] including construct validity and factor structure [59], concurrent validity [60] reliability [61] and test-retest reliability [62] in care home settings. There are also available data on expected change in points from previous similar studies supporting the sample size calculation. The CMAI will be completed in accordance with the *CMAI Manual* [48] via staff proxy report in the context of an interview with a trained researcher.

**Pittsburgh Agitation Scale** [49]:

The PAS is an observational rating of the presence and intensity of agitation within four behaviour groups, which has good reported reliability and validity [49]. Observations are conducted for between 1 and 8 h. In this trial, data collection will be undertaken by an independent researcher using a standardised observation period (between 10.00 and 17.00 hours), on consented residents within communal areas.

**Adapted CMAI** [48]:

The adapted CMAI is a researcher-completed, direct observational tool, which records observed levels of agitation over a single observation period, on a single day. It was adapted specifically for the purpose of this trial since the original CMAI scale considers proxy reported behaviours over the previous 2 weeks and is not suitable for direct observational use on a single day. Adaption included changing the CMAI’s seven-point scale related to the previous 2 weeks to a four-point scale (‘never’ to ‘several times an hour’) appropriate for observations on a single day. In this trial, data collection will be standardised to observations of consented residents within communal areas between 10.00 and 17.00 hours.

##### Behaviours that staff may find challenging to support

**Neuropsychiatric Inventory-Nursing Home (NPI-NH)** [50]:

The NPI-NH is a validated 12-item measure with good reported reliability, that records a range of BSC including, delusions, hallucinations, agitation/aggression, depression/dysphoria, anxiety, elation/euphoria, apathy/indifference, disinhibition, irritability/lability, aberrant motor behaviour, sleep and night-time behaviour disorders and appetite/eating disorders [50]. It will be completed via staff proxy report in the context of an interview with a trained researcher.

Quality of life

Multiple quality of life measures have been implemented since no one measure was identified that could provide the required sensitivity to quality of life in this participant group, and which could facilitate participant self-report in those with severe dementia.

**DEMQOL-Proxy** [63]:

The DEMQOL-Proxy is a quality of life measure with 32 items covering mood, behavioural symptoms, cognition and memory, physical and social functioning and general health that are administered by an interviewer. It is completed by a carer of the person with dementia and administered by an interviewer. It has acceptable psychometric properties for measuring quality of life in dementia [64] and has been valued to enable the derivation of preference based indices (utility values) [65] and will thus be employed in the secondary cost-utility analyses. It will be completed via staff proxy report in the context of an interview with a trained researcher. The relative/friend proxy will also complete the DEMQOL-Proxy where available to provide complimentary additional data.

**EQ-5D-5 L/EQ-5D-5 L Proxy** [53]:

EQ-5D is a standardised measure of health outcome that provides a single index value for health status [66, 67]. It has five items covering: usual activities, self-care, mobility, pain and anxiety/depression, each with five response options (no problems, slight problems, moderate problems, severe problems and unable to do task). It will be completed via staff proxy report in the context of an interview with a trained researcher. The resident will complete the EQ-5D-5 L when able via interview with the researcher and the relative/friend proxy, where recruited, will complete the EQ-5D-5 L Proxy to provide complimentary additional data.

**Quality of Life in Late Stage Dementia (QUALID) [**51**]:**

The QUALID is an 11-item proxy completed scale that rates the presence and frequency of quality of life-related behaviours over the previous 7 days. It is reliable and valid for rating quality of life in people with moderate to severe dementia and has good internal consistency, test-retest reliability and inter-rater reliability. It will be completed via staff proxy report in the context of an interview with a trained researcher. The relative/friend proxy will also complete the QUALID where available to provide complimentary additional data.

**QOL-AD** [68]:

The QOL-AD is a 13-item self-report questionnaire. It has good reported internal reliability, test-retest reliability and convergent validity [68]. It is reported to be reliable for use with people with mild to moderate dementia (11 or greater on the MMSE [69] and more severe dementia (MMSE of >2)) [70, 71]. It will be completed by the resident in the context of an interview with a trained researcher.

##### Use of health care services

Health care resource use measure:

This measure is adapted from one that has recently been piloted in a care home feasibility trial [72]. It captures primary (e.g. general practitioner and nurse visits) and secondary (Accident and Emergency Department and other hospital visits) health care usage. This will be completed by the researcher from care home records.

##### Dementia severity

**Clinical Dementia Rating Scale (CDR)** [73]:

The CDR is a standardised scale for rating the severity of dementia from no cognitive impairment to severe or advanced dementia [74] and is completed by a trained assessor via informal interview/conversation with the person or a proxy. It will be completed by the researcher in discussion with the staff proxy.

**Functional Assessment Staging in Alzheimer’s Disease (FAST)** [42]:

The FAST is a scale designed to capture the functional severity of dementia. It is particularly designed for use in more moderate to severe dementia. It is completed by proxy report from a caregiver. This will be completed by the researcher with information from a staff informant.

#### Staff-related outcome measures

Staff measures are distributed by the researcher or care home manager and returned in a sealed envelope either directly to the researcher or posted into a secure locked box located within the care home during data collection visits. Alternatively, staff may return the measures direct to the CTRU using a pre-paid envelope.

##### Work stress

**General Health Questionnaire 12-item (GHQ-12)** [54]:

The GHQ-12 is a measure of stress/psychological well-being and is used with the general population. It has good reported psychometric properties [75].

##### Job or role efficacy

**Sense of Competence in Dementia care Staff (SCIDS) scale** [55]:

The SCIDS is a self-complete 17-item scale that measures staffs’ sense of their own competence with regard to delivery of dementia care across four sub-scales (professionalism, building relationships, care challenges, sustaining personhood). It has acceptable internal consistency and test-retest reliability [55].

#### Care home-related outcome measures

##### Care quality

**Quality of Interactions Schedule (QUIS)** [56]:

The QUIS is an observational measure of the quality of interactions between staff and people with dementia, which has reported adequate inter-rater reliability and sensitivity [76]. The QUIS is administered via researcher observations, using a time-sampling approach [56, 77]. Data are amalgamated to provide a rating of the quality and quantity of interactions at the care home. In this trial, observations will be undertaken by a researcher for 15-minute periods, in communal areas in the care home, during two 2-h observations (one a.m. and one p.m.) during a 7-day period.

##### Care home environment, context and organisation

**Environmental Audit Tool (EAT)** [78]:

The EAT is a valid and reliable instrument that can be used to differentiate between the quality of environmental design in various types of dementia care facilities [78]. It is completed by the researcher through observation and with information from a senior staff member.

Care home context and organisational questionnaire

Information about the care home context and organisation will be gathered using a specially designed questionnaire asking questions about the home (size, type, ownership, geography, staff turnover, staff ratios, resident demographics, etc.), manager (qualifications, length of time in post, leadership style, etc.) and staff (qualifications, length of time in post, English as first language, etc.) demographics. It is completed by the researcher with information from a senior staff member.

**Group Living Home Characteristics Questionnaire (GLHC)** [79]:

This is a measure of the style of care being delivered in the home. It examines how ‘home-like’ the care delivered is. It is completed by the researcher with information from a senior staff member.

### Bias and blinding

Due to the nature of the intervention, it will not be possible to blind care homes or staff to the allocation status. To minimise the potential for bias, the trained mappers will not be involved in providing any outcome data. To ensure consistency, where possible the same staff member will be asked to complete resident measures at each data collection point. All data will be gathered by trained researchers in discussion with the informant with the exception of the staff measures questionnaires, which will be completed independently by staff. To ensure reliability and to restrict the potential for researcher bias, intra-rater reliability will be achieved for all researchers at training. Effort will be made to blind all trial researchers to allocation status. The researcher conducting the observations for the PAS and abridged CMAI will be independent and will remain blind. Any unblinding will be recorded and unblinded researchers will not conduct any further follow-up data collection in those care homes.

### Sample size

The sample size calculations were based on assumptions of an average of 40 residents in each care home, and that at least 60 % of these would meet the eligibility criteria and 65 % of those eligible would be willing to provide informed consent (i.e. 15 residents recruited from each care home). Calculations were based on a normally distributed outcome: the mean difference in CMAI scores between arms at 16 months. Fifty care homes, each recruiting 15 residents, will result in 750 residents overall and provide 90 % power to detect a clinically important difference of 3 CMAI points (standard deviation (SD) 7.5 points) with a two-sided 5 % significance level. This allows for 25 % loss to follow-up (cluster size of 11 residents available for analysis) based on Chenoweth et al. [14] leading to an inflation factor of 2.0 (intracluster correlation coefficient (ICC) no greater than 0.1). The assumption that the ICC will be no larger than 0.1 was based on an ICC for CMAI reported by Fossey et al. [27] when evaluating effectiveness of a psychosocial intervention on antipsychotic use in nursing home residents with dementia.

### Methodological improvements building on previous studies

Through correspondence with the authors of all three previous trials on the efficacy of DCM™, we were able to identify key study design strengths, challenges and difficulties encountered. Key methodological developments in the EPIC trial include:

- Adoption of a pragmatic trial design. This enables generalisation of the treatment effects to practice in the UK and important data to be collected on cost-effectiveness
- Use of an independent researcher assessment of the primary outcome at each time point, permitting assessment of any bias that may be caused by being unable to blind study participants to intervention allocation
- Collection of quality of life data from three sources where available – staff-proxy, self-report and relative proxy
- Collection of quality of life data using three measures, permitting, use of measures we believe to be most sensitive for use in this population (QUALID), quality-adjusted life years (QALYs) to be calculated (DEMQOL-Proxy) and self-report in as many residents as possible (QOL-AD)
- Randomisation of sites at a care home rather than unit level so there is a significantly reduced chance of control contamination in care home sites where one or more units may be participating but may be randomised to the intervention separately
- Inclusion of a process evaluation including in-depth intervention fidelity assessment and qualitative examination of implementation issues

### Statistical analysis

A single final analysis is planned when all follow-up data have been collected and the primary analyses will be carried out on an intention-to-treat basis, utilising all available follow-up data, comparing treatments as allocated.

It is expected that a sizeable proportion of residents will be missing from the main analyses, and that missing data can be predicted by known variables, hence the principal method for handling missing scale data will be multiple imputation under the Missing at Random (MAR) assumption. Sensitivity analyses will be carried out to assess the impact of the choice of imputation model and of assuming data are Missing Not at Random (MNAR) as appropriate.

The impact of cluster randomisation is expected to be equal across arms but that of treatment provision is not. As such, the principal method for handling clustering effects will be to fit a multilevel model that allows care home- and resident-level variances to differ across arms. A sensitivity analysis will be conducted fitting a random intercept model assuming equal total variances. The resident-level primary outcome of agitation (continuous CMAI score) will be analysed at 16 months post randomisation using a linear two-level heteroscedastic regression model [70], adjusting for design factors, with a contrast for intervention and control. The model will be adjusted for the following fixed effects: care home (level 2) covariates (home type and size, provision of dementia awareness training and hub) and resident (level 1) covariates (severity of dementia, age and baseline CMAI score). Unadjusted and adjusted ICCs, treatment effect estimates and corresponding 95 % confidence intervals will be presented.

For residents where a PAS and an abridged CMAI score are also available, a sensitivity analysis will be conducted replacing the CMAI score in the primary analysis with the PAS and abridged CMAI scores looking for consistency in the size and direction of effect.

Secondary outcome measures will be analysed using a similar modelling strategy as described for the primary analysis. Where outcomes are continuous, linear models will be fitted; where binary, logistic models will be fitted. Change in primary and secondary outcomes over time (6 and 16 months) will be analysed with three-level multilevel models with contrasts for treatment, time and the treatment-by-time interaction, in which outcomes are nested within residents and care homes. A similar correlation structure will be assumed for care homes and residents, but correlation over time will also be considered at the outcome level.

A number of exploratory subgroup analyses are planned which will be specified in detail in the Statistical Analysis Plan. These will include care home- and resident-level factors such as type of care home, severity of dementia and NPI subgroup clusters.

### Health economic evaluation

The proposed primary endpoint and methods for the economic evaluation follow the reference case set out by the National Institute for Health and Care Excellence (NICE) [80]. The primary economic analysis will be a cost-utility analysis over 16 months presenting incremental cost-effectiveness ratios (ICER) for intervention (UC + DCM™) versus control (UC), with effects expressed in terms of quality-adjusted life years (QALY). The analysis will adopt the health care and personal social services perspective. Analysis of the uncertainty surrounding the ICER will be undertaken using non-parametric bootstrap simulation (10,000 simulations) and presented on a cost-effectiveness plane and a Cost-Effectiveness Acceptability Curve (CEAC) [81]. A net benefit regression approach will also be employed using the model selected for the clinical effectiveness analysis [82]. Where Net Monetary Benefit (NMB) = (£20,000 × QALYs) − Costs NMB regression will enable covariate control and clustered analysis. There will be no modelling forward of benefits and discounting (at 3.5 %) will be conducted for values post 12 months. We will use the NICE willingness to pay per incremental QALY threshold range (lambda [λ] = £20,000-£30,000) to determine cost-effectiveness.

#### Effects

Utility values will be captured using the EQ-5D-5 L (primary) and DEMQOL-Proxy-U [83] (secondary). Residents will self-report where able on the EQ-5D-5 L or the EQ-5D-Proxy will be used. Resident- and proxy-reported data will be reported and analysed separately. However, given that only a proportion of residents will be able to complete the questionnaires, we will explore whether it is valid to use one source of data as a substitute for the other.

#### Costs

The total cost of DCM™ will incorporate the costs of training staff and staff time spent delivering the intervention as well as travel costs and any other expenditure (e.g. on training materials). The assumption for the analysis will be that the local authority pays for the provision of care home care for residents. We will include a sensitivity analysis where a proportion of residents are considered to pay toward their care home costs. Researchers will collect health care resource use data for each resident participating in the trial at baseline, 6 and 16 months using individual care home records and care plans. This will be supplemented by care home-level data collection, which will enable some validation of individual-level data. Unit costs for health service staff and resources will be obtained from national sources such as the Personal Social Services Research Unit, the *British National Formulary* and NHS reference cost database.

### Trial governance

The Trial Management Group, comprising the chief investigator, CTRU team, co-investigators and researchers will be assigned responsibility for the clinical set-up, on-going management, promotion of the trial, and for the interpretation of results. An independent Data Monitoring and Ethics Committee will be established to review the safety and ethics of the trial. An independent Trial Steering Committee will be established to provide overall supervision of the trial, including trial progress, adherence to protocol, resident safety, and consideration of new information. The trial sponsor will ensure responsibility and accountability for trial conduct and procedures associated with the protocol. Individual care homes remain responsible for participant care as usual. Trial researchers will have the opportunity to highlight any safeguarding issues of concern with the Trial Steering Committee and to individual care homes, in line with relevant guidance from the local authority, care home, and the trial team.

### Public and Patient Involvement

Service users and carers will play an integral role throughout this programme of research to ensure that the work is based on the principles of Patient and Public Involvement (PPI). PPI will be fulfilled in partnership with the Alzheimer’s Society via involvement of members of their Research Network, and via inclusion of PPI representatives (relatives and persons with Dementia) on the Trial Management Group and Trial Steering Committee. Specifically, PPI representatives will be involved in the review of participant information and general aspects of trial design and be involved in all decisions made by the Trial Management Group. The intention is to involve service users in the interpretation of results and appropriate dissemination of information at the end of the trial.

## Discussion

### Pilot phase

A pilot was built into the trial to pilot trial procedures to inform modification to, and streamlining of, research processes for the remaining care homes. Two care homes (one in West Yorkshire, one in London), were recruited to the pilot 2 months ahead of start of the planned recruitment phase. Three review periods followed: the first focussed on care home screening and recruitment processes, the second assessed trial burden to participants, and the third adherence to determine if data collection needed to be adapted or reduced. The trial procedures described above were followed, and experiences reviewed by the trial team. The changes made are summarised below. Pilot homes will be included in the final analysis.

#### Screening and recruitment review

One change made as a result of this review was improvement to the Case Report Forms (CRFs). Changes to the content and structure of some CRFs and creation of new forms ensured that all CRFs reflected actual processes and procedures that the researchers needed to follow to accurately complete the complex screening and recruitment process. A second change was made to care home eligibility criteria. Originally, the criteria stated that to be eligible a care home should have a minimum of 24 permanent residents of whom at least 60 % were estimated to have dementia. However, this did not always ensure that recruited care homes would be able to provide an adequate number of trial participants. For example, large residential homes where the general resident population did not include many people with dementia, but contained a smaller specialist dementia unit failed to reach the threshold of 60 % of residents with dementia within the care home overall but could still achieve a viable cluster size of residents with dementia within the specialist unit. Likewise, in smaller homes, an adequate cluster of eligible residents was not always available when both the 24 permanent residents and the ‘60 % of residents with dementia’ criteria were met, due to eligibility exclusions based on other, resident-level, criteria. This was particularly the case for nursing homes where residents had a range of significant additional physical health problems. On the other hand, the criteria also led to the exclusion of some smaller dementia-specialist care homes that would have been able to provide an adequate cluster size despite having less than 24 permanent residents in total. As a result, care home eligibility criteria were replaced with the following:

• Has a sufficient number of permanent residents with dementia (based on a formal diagnosis or Functional Assessment Staging of Alzheimer’s Disease (FAST) score of 4+) eligible to participate in the study in order to achieve a minimum of 10 residents registered to take part prior to care home randomisation

#### Burden review

The burden review was undertaken shortly after the return of the baseline measures. Researchers and relatives/friends were asked to provide details of the time taken to collect each of the measures. A review of the mean, mode and range of time taken to complete each questionnaire was completed (see Table 2). The number of returned relative/friend measures was low and the data on time taken to completion was varied and inconsistent. Therefore, it was not possible to reach a meaningful conclusion regarding the data collection burden on relatives/friends based on the pilot review alone.

**Table 2:** Summary of burden assessment

Questionnaire	Completed by	Number of data points	Mean time taken (min)	Modal time taken (min)	Range (min)
CMAI	Staff proxy	24	07	10	(02–15)
NPI-NH	Staff proxy	16	15	20	(08–24)
FAST	Staff proxy	24	04	03	(02–14)
CDR	Staff proxy	23	06	05	(03–12)
DEMQOL	Staff proxy	24	09	05	(04–17)
EQ-5D	Staff proxy	24	03	03	(02–07)
QUALID	Staff proxy	24	07	07	(04–20)
Total	Staff proxy		53	67	(34–74)
EQ-5D	Resident	21	06	05	(02–28)
QOL-AD	Resident	22	13	10	(05–20)
Total	Resident		18	15	(12–30)
Visit details	Relative/friend	8	02	01	(01–06)
DEMQOL	Relative/friend	9	10	10	(01–30)
EQ-5D	Relative/friend	9	04	05	(01–12)
QUALID	Relative/friend	9	08	05	(01–20)
Total	Relative/friend		26	22	(04–68)
Current comorbidities	Researcher	24	05	02	(02–15)
Resource Use Form	Researcher	22	10	10	(05–20)
Total	Researcher		15	15	(07–30)

*CDR* Clinical Dementia Rating Scale, *CMAI* Clinical Dementia Rating Scale, *DEMQOL* Dementia Quality of Life measure, *EQ-5D* EuroQol five dimensions, *FAST* Functional Assessment Staging of Alzheimer’ Disease, *NPI-NH* Neuropsychiatric Inventory-Nursing Home, *QUALID* Quality of Life in Late-Stage Dementia, *QOL-AD* Quality of Life in Alzheimer’s disease measure

The burden placed on staff completing proxy measures was of initial concern, given the upper completion time of 74 minutes. However, the researchers advised that proxy measures could be completed with a staff member in multiple sessions of 15–20 min over one or more days and longer completion times were often associated with staff choosing to provide detailed responses to measure items. Therefore, given the necessity of each of the individual measures within the trial, it was not possible to identify a suitable way to reduce this burden in any meaningful way.

#### Adherence

Only two residents of a possible 26 (7.7 %) did not complete self-report measures; one declined to answer any of the item questions at all and one stopped half way through the administration of the first questionnaire. It was judged that the burden on the residents was not overly demanding and that the measures used were likely to be completed by a majority of trial participants. Therefore, no changes were made to the number of measures administered with residents. The researchers identified two questions in the QOL-AD that appeared to be causing anxiety/distress to some residents and were leading to missing data on these items. The items related to a question about satisfaction with management of their own finances (which the resident may no longer be in control of) and relationship with the residents spouse (who may no longer be alive). Thus, a modified version of the QOL-AD [84] specifically designed for use with people with dementia in care homes was identified and implemented. It excludes the two questions causing distress and includes four additional questions on relationships with staff, ability to live with others, taking care of yourself and the ability to make choices in your life.

Other changes to the protocol implemented following the pilot include a modification to the randomisation stratification criteria which originally included ‘use of DCM™ in the home over the last 18 months to 5 years’ and replacing it with ‘recruitment hub’ to ensure spread of intervention homes across the three hubs, removing a possible regional effect. The staff measures collection process has been adapted to include the option for staff to post their questionnaires directly back to the CTRU, as some staff stated they felt uncomfortable leaving their replies in the care home where other members of staff might have access to them. Lastly, an additional process of reminding intervention homes of an upcoming mapping cycle by text message was implemented, in addition to sending of newsletters and intervention paperwork via the post, to increase potential intervention implementation.

To conclude, the pilot proved valuable for the researchers to implement proposed trial processes and documentation and to suggest adaptations, which have been implemented in a timely manner. This has been important in ensuring smooth running of the trial once recruitment rates have increased.

## Trial status

This is the protocol of a current trial; recruitment of participants is on-going.

## Additional notes

A Standard Protocol Items: Recommendations for Interventional Trials (SPIRIT) checklist is included for this protocol (see Fig. 1).

## Trial sponsor

Leeds Beckett University

University Research Office

James Graham Building

Headingley Campus

Leeds Beckett University

Leeds

LS6 3QS

## Abbreviations

BSC, behaviours that staff may find challenging to support; CDR, Clinical Dementia Rating Scale; CMAI, Cohen-Mansfield Agitation Inventory; CRF, Case Report Form; CTRU, Clinical Trials Research Unit; DCM™, Dementia Care Mapping™; DEMQOL-Proxy, Dementia Quality of Life measure – proxy version; EAT, Environmental Audit Tool; FAST, Functional Assessment Staging of Alzheimer’s Disease; GHQ-12, General Health Questionnaire – 12-item; GLHC, Group Living Home Characteristics; ICC, intracluster correlation coefficient; ICER, Incremental cost-effectiveness ratio; MAR, Missing at Random; MNAR, Missing Not at Random; NICE, National Institute for Health and Care Excellence; NMB, Net Monetary Benefit; NPI, Neuropsychiatric Inventory; PAS, Pittsburgh Agitation Scale; PPI, Patient and Public Involvement; QALY, quality-adjusted life year; QOL-AD, Quality of Life in Alzheimer’s disease measure; QUALID, Quality of Life in Late-Stage Dementia; QUIS, Quality of Interactions Schedule; RCT, randomised controlled trial; SAE, serious adverse event; SCIDS, Sense of Competence in Dementia Care Staff scale; UC, Usual Care

## Additional file

Additional file 1: Schedule of enrolment, interventions and assessments. (PDF 122 kb)
