# Supplementary material for: Evaluating the effectiveness and cost-effectiveness of Dementia Care Mapping™ to enable person-centred care for people with dementia and their carers (DCM-EPIC) in care homes: study protocol for a randomised controlled trial
Source: Trials. 2016 Jun 24;17:300. doi: 10.1186/s13063-016-1416-z (PMC4921015; doi:10.1186/s13063-016-1416-z)
Supplement: Additional file 1: — Schedule of enrolment, interventions and assessments. (PDF 122 kb) [file 13063_2016_1416_MOESM1_ESM.pdf]

	Study Period																				
	CH Screening	Enrolment	Training	Participant Recruitment	Allocation	Post Allocation															
TIMEPOINT					0	1	2	3	4	5	6	7	8	9	10	11	12	13	14	15	16
Enrolment:																					
CH Eligibility Screening	X																				
CH Invitation to Participate	X																				
CH Informed Consent		X																			
Training Review		X																			
Dementia Awareness Training			•—	—•																	
Resident Screening				X																	
Participant Eligibility (Resident/Proxy Informant/Staff/Mapper)				X							X										X
Participant Recruitment (Resident/Proxy Informant/Staff/Mapper)				X																	
Resident Registration				X																	
CH Randomisation					X																
Interventions:																					
Usual Care						•—	—	—	—	—	—	—	—	—	—	—	—	—	—	—	—•
Dementia Care Mapping								X					X					X			
Assessments:																					
CMAI (Cohen Mansfield Agitation Index)				X							X										X
PAS (Pittsburgh Agitation Scale)				X							X										X
Care Home Manager Demographics				X							X										X
Care Home Demographics				X							X										X
GLHC (Group Living Home Characteristics)				X							X										X
EAT (Environmental Audit Tool)				X							X										X
QUIS (Quality of Interactions Schedule)				X							X										X
Staff Proxy Informant Demographics				X							X										X
Resident Mental Capacity Check				X							X										X
Resident Demographics				X							X										X
RF Proxy Informant Demographics				X							X										X
NPI-NH (Neuropsychiatric Inventory)				X							X										X
FAST (Functional Assessment Staging)				X							X										X
CDR (Clinical Dementia Rating)				X							X										X
DEMQOL Proxy				X							X										X
EQ 5D 5L				X							X										X
QUALID				X							X										X
QOL-AD				X							X										X
Resident Comorbidities				X							X										X
Healthcare Resource Use				X							X										X
Prescription Medications				X							X										X
Staff Demographics				X							X										X
SCIDS (Sence of Competence in Dementia care Staff)				X							X										X
Safety Reporting (Hospitalisations)						•—	—	—	—	—	—	—	—	—	—	—	—	—	—	—	—•
Qualitative Interviews (Process Evaluation)																					X

* If required - based upon Training Review
